# Measuring the Evolution of Ontology Complexity: The Gene Ontology Case Study

**DOI:** 10.1371/journal.pone.0075993

**Published:** 2013-10-11

**Authors:** Olivier Dameron, Charles Bettembourg, Nolwenn Le Meur

**Affiliations:** 1 Université de Rennes, Rennes, France; 2 Institut de Recherche en Informatique et Systèmes Aléatoires, Rennes, France; 3 UMR1348 PEGASE Institut national de la recherche agronomique – Agrocampus OUEST, Rennes, France; 4 Ecole des hautes études en santé publique, Rennes, France; University of Lausanne, Switzerland

## Abstract

Ontologies support automatic sharing, combination and analysis of life sciences data. They undergo regular curation and enrichment. We studied the impact of an ontology evolution on its structural complexity. As a case study we used the sixty monthly releases between January 2008 and December 2012 of the Gene Ontology and its three independent branches, i.e. biological processes (BP), cellular components (CC) and molecular functions (MF). For each case, we measured complexity by computing metrics related to the size, the nodes connectivity and the hierarchical structure.

The number of classes and relations increased monotonously for each branch, with different growth rates. BP and CC had similar connectivity, superior to that of MF. Connectivity increased monotonously for BP, decreased for CC and remained stable for MF, with a marked increase for the three branches in November and December 2012. Hierarchy-related measures showed that CC and MF had similar proportions of leaves, average depths and average heights. BP had a lower proportion of leaves, and a higher average depth and average height. For BP and MF, the late 2012 increase of connectivity resulted in an increase of the average depth and average height and a decrease of the proportion of leaves, indicating that a major enrichment effort of the intermediate-level hierarchy occurred.

The variation of the number of classes and relations in an ontology does not provide enough information about the evolution of its complexity. However, connectivity and hierarchy-related metrics revealed different patterns of values as well as of evolution for the three branches of the Gene Ontology. CC was similar to BP in terms of connectivity, and similar to MF in terms of hierarchy. Overall, BP complexity increased, CC was refined with the addition of leaves providing a finer level of annotations but decreasing slightly its complexity, and MF complexity remained stable.

## Introduction

### The problem of ontology quality variation

Ontologies are instrumental for sharing, combining and analyzing life sciences data [1]. Ontologies evolve through regular modifications related to curation or to enrichment [2]. Existing metrics quantifying the changes rely on the variation of the number of classes, of the number of properties, or for the most sophisticated, of the number of restrictions [3]. For example, the Ontology Evolution Explorer OnEX provides access to approximately 560 versions of 16 life science ontologies. It allows a systematic exploration of the changes by generating evolution trend charts and inspection of the added, deleted, fused and obsolete concepts [4]. The underlying assumption of these approaches is that for ontologies, the more classes and properties, the better.

However, the creation of a new class could decrease the overall quality of the ontology, whereas previous measures would increase. Likewise, deleting an erroneous class would increase the overall quality of the ontology, but previous measures would decrease. Moreover, these measures are not affected if one class is moved from one location to another, nor if one class is deleted and another one added.

### Related general approaches

Together with OnEX, GOMMA is a generic infrastructure for managing and analyzing life science ontologies and their evolution [3]. It provides advanced comparison capabilities of two versions of an ontology. Its Region Analyzer identifies evolving and stable regions of ontologies by determining the cost of different change operations such as deletions and additions.

Malone and Stevens measured the activity of an ontology by analyzing the additions, deletions and changes as well as the regularity and frequency of releases [5] on 5036 versions of 43 ontologies. They successfully identified five profiles of activity (initial, expanding, refining, optimizing and dormant).

While the previous two approaches focused on changes by analyzing ontology variations, others took a static perspective on ontology analysis. OntoClean is a formal method for structuring and analyzing ontologies based on metaproperties of classes (identity, unity, rigidity and dependence) [6]. To our knowledge, there is no effort to apply this method to the GO. Kölher et al. developed the GULO (Getting an Understanding of LOgical definitions) Java package for automatic reasoning on classes logical definitions [7]. Its exploits the logical definitions and the explicit cross-references between ontologies to compare the relations in the ontology of interest with relations inferred from the references ontologies. This facilitates the systematic detection of omissions and incompatibilities. Shchekotykhin et al. proposed an entropy-based approach for localizing faults when debugging ontologies [8]. Yao et al. formally defined metrics of an ontology's fit with respect to published knowledge in the form of other ontologies and of scientific articles [9]. Hoehndorf et al. propose a method to evaluate biomedical ontologies for a particular problem by quantifying the success of using the ontology for this problem [10]. Comparing the measures of success of two versions of an ontology for the same problem would provide an indication of the relevance of the modifications.

These generic solutions were completed by various ontology-specific efforts to detect inconsistencies or ambiguities, such as the Unified Medical Language System (UMLS) [11], the Medical Entities Dictionary [12], the Cancer Biomedical Informatics Grid (CaBIG) [13], the NCI Thesaurus (NCIt) [14]. Other approches relied on the ontology structure, e.g. for the Foundational Model of Anatomy (FMA) [15] or on logical definitions of classes, e.g. on the Cell Ontology [16] or SNOMED-CT [17].

Yao et al. provide a review of ontology evaluation and identified four categories: (1) measures of an ontology's internal consistency, (2) usability and task-based performance, (3) comparison with other ontologies and (4) match to reality [9].

### Ontology complexity as a measurable proxy for ontology quality

There is a need for a finer grain measure of the quality of an ontology which would allow a better assessment of the impact of a change or of a set of changes. One of the difficulties of defining and measuring the quality of an ontology is that it refers to how well the ontology reflects reality, of which we have an incomplete and imperfect understanding. Ontology complexity is an aspect of quality more amenable to formal analysis. Moreover, it focuses on an intrinsic feature of an ontology, not its suitability for a particular task.

None of the previous general efforts addresses the question of the impact of the changes on the ontology complexity. We propose an approach based on ontology complexity. Compared to Yao et al.'s four categories of ontology evaluation [9], it offers a complementary view but is different from ontology's internal consistency.

### Measures of ontology complexity

As a test-case, we focus on the Gene Ontology (GO). This ontology is one of the most widely used and actively maintained in the biomedical domain [18]. Among the keys of its success are its continuous evolution and its active curation [19]. Recent efforts focused on improving the modeling of apoptosis and cardiac conduction, and on increasingly using the Web Ontology Language OWL in the GO infrastructure, which in turn supports TermGenie (http://go.termgenie.org/) to automatically place terms in the hierarchy [20].

We investigated whether GO structural complexity increased monotonously over the last five years, as did its size. We focused on the study of nodes' connectivity and of the graph's hierarchy, based mostly on the subsumption relation. In the discussion, we compare our approach to other works focusing on GO evolution.

## Resources and Methods

### Structure of the gene ontology

The Gene Ontology is a collaborative effort to deliver a species-independent uniform vocabulary for describing gene products [18]. Its classes, also called “GO terms” are organized in three separate branches describing gene products' molecular functions (MF), the biological processes (BP) they participate in and their location in cellular components (CC).

GO also recognises that these classes can have different granularities, i.e. different levels of precision, or be connected by several relations. It organizes them as a directed acyclic graph that supports reasoning (http://www.geneontology.org/GO.ontology.relations.shtml).

Within each branch, the classes are connected by three kinds of relations. The classes are organized in a taxonomy with occasional multiple inheritance along the is a relation which connects a subclass to its superclass (for example, “Carbohydrate metabolic process” (GO:0005975) is a subclass of both “Organic substance metabolic process” (GO:0071704) and “Primary metabolic process” (GO:0044238)). The part of relation connects a part to a whole (for example, “Golgi cisterna” (GO:0031985) is a part of “Golgi stack” (GO:0005795)). The regulates relation connects a regulator process to a regulated process (for example, “Regulation of meiosis” (GO:0040020) regulates “Meiosis” (GO:0007126)). Contrary to the is a and part of relations, regulates has two more specific subrelations: positively regulates and negatively regulates. This leads to a systematic modeling pattern where each regulation process has two subclasses representing the positive and negative regulation processes (the subclasses of “Regulation of meiosis” (GO:0040020) are “Positive regulation of meiosis” (GO:0045836) and “Negative regulation of meiosis” (GO:0045835)), and each of them is connected to the process they regulate (here, “Meiosis” (GO:0007126)) by either regulates, positively regulates or negatively regulates.

### Successive gene ontology versions

We retrieved the 60 successive Gene Ontology monthly releases between January 2008 and December 2012 in the OBO format from the Gene Ontology archives (files gene_ontology_edit.obo.2008-01-01.gz to gene_ontology_edit.obo.2012-12-01.gz at http://www.geneontology.org/ontology-archive/).

Each of them was converted to the OWL format using Protégé (http://protege.stanford.edu/).

The January and February releases from 2009 appeared to be identical. A personal communication with the Gene Ontology support team confirmed the error and pointed to revision 5.930 from January 31, 2009 from the CVS repository (http://cvsweb.geneontology.org/cgi-bin/cvsweb.cgi/go/).

The January 2012 monthly release was not generated. We replaced it by the daily release, which had not changed between 24th December 2011 to 3rd January 2012.

### Methods

In order to characterize the evolution of the GO complexity from January 2008 to December 2012, we followed a four-step approach. First, we studied the evolution of the number of classes and relations as a baseline. This gave global indications on the size of the graph. Second, we used several directed acyclic graph (DAG) metrics reflecting the nodes connectivity. This gave local indications on the nodes. Third, we used tree and directed graph hierarchy-related metrics reflecting the graph topological structure. This gave global indications on the ontology semantics. Fourth, we controlled whether our metrics are able to tell the difference between the real modifications as observed between two successive versions of the ontology, and some random modifications. The idea is that failing to do so would question the relevance of the metrics. We used the February 2010 version of the GO as a baseline. We compared randomly-generated ontology modifications with the March 2010 version in order to study whether or not the previous metrics could discriminate randomly-generated ontology modifications from genuine ones.

During our study, we considered the Gene Ontology both as a whole and by distinguishing its three branches: BP, CC and MF. The branches had different relative sizes. In December 2012, BP represented approximately 66% of the total number of classes, CC represents 8% and MF 26%. The rationale was to detect if some variations of one branch were compensated by some other branch, and to determine if the evolution of the Gene Ontology was uniform among BP, CC and MF.

The modeling pattern for representing process regulation results in each positive or negative regulation relation being systematically subsumed by a regulates relation at the superclass level. In order to avoid counting relations multiple times, we only considered the regulates relation.

### Complexity metrics

In this section, we define the graph metrics used to study the evolution of size, connectivity and of topology of GO. Throughout the paper, we used “metrics” to refer to a formula, and “measure” to refer to the value of a metrics. Table 1 summarizes the formal definitions for the metrics used in the first three steps. We adapted the generic framework proposed by Hartung et al. to study the structural changes occurring within ontologies [2]. An ontology modeled as a directed graph is represented by a pair 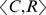 where 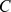 is the set of the classes of the ontology (the nodes of the graph), and 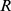 is the set of the typed relations between the classes (the edges). 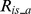 is the set of the is a relations between classes (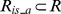). Similarly, 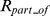 and 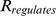 represent the sets of part of and regulates between the classes (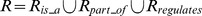).

**Table 1:** Ontology complexity metrics.

Ontology aspect	Metrics	Scope	Definition
Size	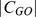	Global	Number of classes in GO
	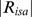	Global	Number of is a relations in GO
	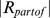	Global	Number of part of relations in GO
	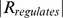	Global	Number of regulates relations in GO
Connectivity	Average degree	Local	2*( 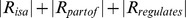 )/( 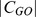 )
	Av. nb is a	Local	( 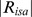 )/( 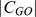 )
	Av. nb part of	Local	( 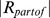 )/( 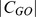 )
	Av. nb regulates	Local	( 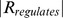 )/( 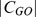 )
Hierarchy	Proportion of leaves	Global	(Nb of classes with no subclasses)/( 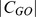 )
	Av. height	Local	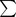 (max length of path from node to leaf)/( 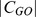 )
	Av. depth	Local	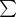 (max length of path from node to root)/( 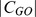 )

Description of the metrics used to quantify the complexity variations of an ontology. The definitions are given for GO and can be adapted to BP, CC and MF.

The size of an ontology depends on its number of classes and its number of relations. 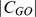 represents the number of classes in GO. Likewise, 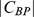, 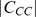 and 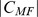 represent the respective numbers of classes of the BP, CC and MF branches. 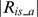 represents the number of is a relations in GO. Similarly, 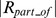 and 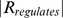 represent the respective numbers of part of and regulates relations. Branch-specific variations such as 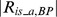 representing the number of is a relations in BP are defined similarly.

Connectivity measures differentiate a sparse graph from a complete graph. The degree of a node is the number of nodes it is directly connected to. Comparing the successive values of the average degree indicated if the graph became more sparse of more dense regardless of the evolution of its size. We used degree-related metrics such as the average number of is a, part of and regulates relations to examine these relations contributions to the average degree.

Ontologies are not only directed acyclic graphs. They also follow a principled hierarchical organization based on the is a relation. Throughout the paper, we used “graph topology” when refering to relations in general, and “graph hierarchy” when refering to metrics taking the semantics into account. In the evolution of an ontology we expect classes to be added at each levels of the hierarchy: close to the root, in the middle, and as leaves (i.e. classes that have no subclasses). Because of inheritance, modifications of an is a relation between two nodes has remote consequences on their descendants and ancestors. To reflect this principled organization of an ontology, we used several hierarchy-related metrics. We computed the proportion of leaves, and nodes' average height and average depth. The height of a node is the maximum length of the paths from a leaf to this node. It represents how far a node is from the leaves. The depth of a node is the maximum length of the paths from this node to a root. It represents how far a node is from the root.

### Generation and analysis of the random ontologies

We studied if the previous metrics could discriminate randomly-generated ontology modifications from genuine ones. Based on the February 2010 version of GO, we generated fifty simulated ontologies by adding randomly the same numbers of classes and relations. The proportions were respected for BP, CC and MF (e.g. there were 395 classes added to BP in March 2010, so we randomly added 395 classes to BP in each of the fifty simulated ontologies). For each simulation and for BP, CC and MF separately, we created the classes to be added and randomly selected a parent for each of them (thus generating as many random is a relations as classes to be added). We then created the remaining random is a relations, and the random part of and regulates relations. Note that a random class can be created as a subclass of another previous random class, forming a new branch of the hierarchy.

We compared the simulated values with the value observed in March 2010 for average depth, average height and proportion of leaves. The null hypothesis was “There is no statistically significant difference between the measured values of the randomly-generated ontologies and the value observed between the February and March 2010 version of GO”. We performed two-sided Student's t-tests with an 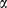 parameter of 0.05 using R version 3.0.0.

## Results

Spreadsheets containing the results are available as supplementary files.

S1-geneOntology-complexityEvolution-monthly.ods contains the analysis of the sixty Gene Ontology monthly releases between January 2008 and December 2012.

S2-geneOntology-enrichmentSimulations.ods contains the analysis of the fifty simulated random ontologies.

### Variations of number of classes and relations

**Figure 1:**
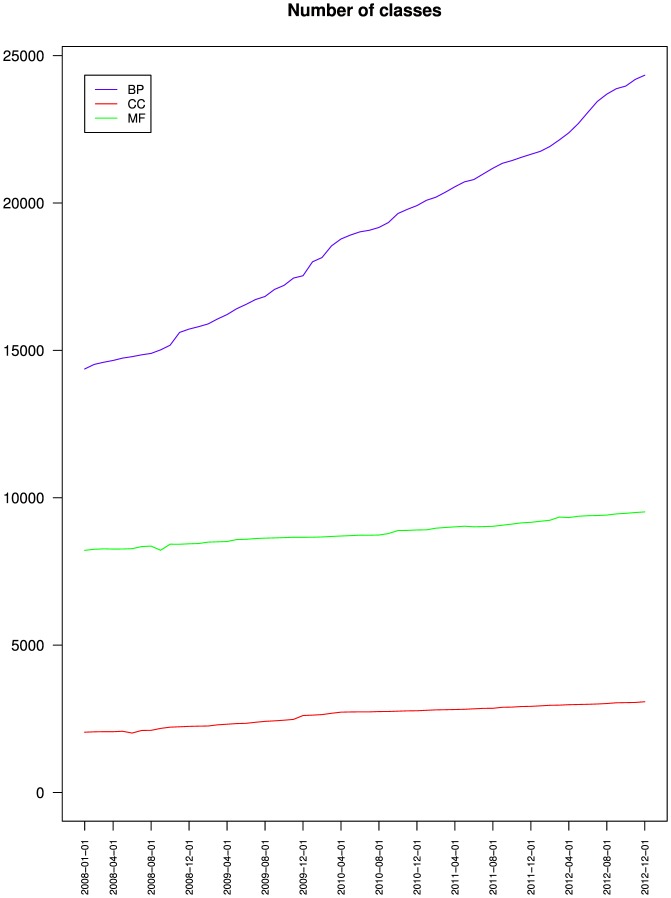
Evolution of the number of classes of the three branches of the Gene Ontology. Biological process (BP), Cellular component (CC) and Molecular function (MF).

**Figure 2:**
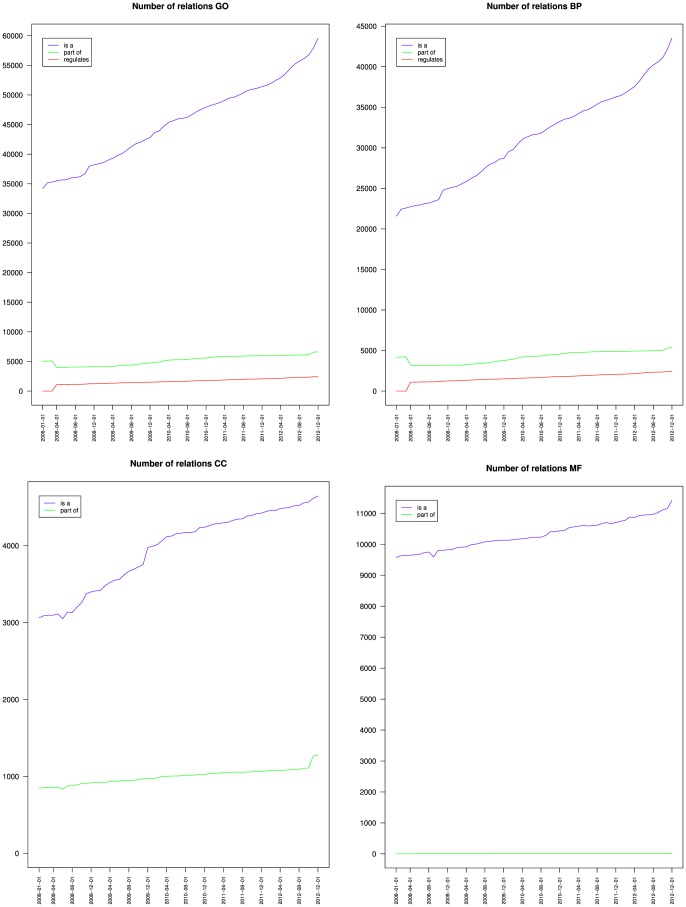
Evolution of the number of relations of the Gene Ontology (top left) and its Biological process (top right), Cellular component (bottom left) and Molecular function (bottom right) branches.

Figure 1 and Figure 2 show that the number of classes and of relations increased monotonously but at different rates during the time of study.

Table 2 shows that the number of classes increased by 50% for GO, 69% for BP, 51% for CC and 16% for MF between January 2008 and December 2012. These different growth rates modified the relative importance of the three branches. Over the study period, Table 3 shows that the proportion of BP classes increased from 58% to 66% of the Gene Ontology, stayed around 8% for CC and decreased from 33% to 26% for MF. Meanwhile, the number of relations increased by 85% for GO, 115% for BP, 51% for CC and 16% for MF. Table 4 shows that the proportion of BP relations increased from 66% to 76% of the Gene Ontology and decreased from 10% to 8% for CC and from 24% to 16% for MF.

**Table 2:** Gene Ontology complexity variations.

	BP			CC			MF		
	Jan 2008	Dec 2012	%	Jan 2008	Dec 2012	%	Jan 2008	Dec 2012	%
Nb. classes	14,369	24,335	+69.36%	2,046	3,080	+50.54%	8,216	9,520	+15.87%
Nb. relations	25,719	55,341	+115.18%	3,908	5,919	+51.46%	9,583	11,430	+19.27%
Nb. is a	21,563	43,524	+101.85%	3,062	4,647	+51.76%	9,581	11,421	+19.20%
Nb. part of	4,156	5,323	+28.08%	846	1,272	+50.35%	2	9	+350.00%
Nb. regulates	0	2,429		0	0		0	0	
Av. degree	3.58	4.55	+27.05%	3.82	3.84	+0.61%	2.33	2.4	+2.94%
Av. is a	1.5	1.79	+19.18%	1.5	1.51	+0.81%	1.17	1.2	+2.88%
Av. part of	0.29	0.22	–24.37%	0.41	0.41	–0.12%	2.43E^−4^	9.45E^−4^	+288.36%
Av. regulates	0	0.1		0	0		0	0	
Prop. leaves	0.55	0.53	–3.24%	0.76	0.78	+2.71%	0.8	0.8	–0.35%
Av. depth	6.22	7.29	+17.16%	4.97	4.79	–3.46%	5.50	5.62	+2.20%
Max. depth	13	15	+23.08%	10	10	0%	14	15	+7.14%
Av. height	0.89	0.97	+9.19%	0.45	0.40	–11.86%	0.36	0.37	+3.36%

Proportional variations of ontology metrics for Biological process (BP), Cellular components (CC) and Molecular functions (MF) between January 2008 (reference) and December 2012.

**Table 3:** Proportions of classes for the three Gene Ontology branches.

	BP		CC		MF	
	Classes	% GO	Classes	% GO	Classes	% GO
Jan 2008	14,369	58.34%	2,046	8.31%	8,216	33.36%
Dec 2012	24,335	65.89%	3,080	8.34%	9,520	25.78%

Proportions of total number of Gene Ontology classes for Biological process (BP), Cellular components (CC) and Molecular functions (MF) between January 2008 and December 2012.

**Table 4:** Proportions of relations for the three Gene Ontology branches.

	BP		CC		MF	
	Relations	% GO	Relations	% GO	Relations	% GO
Jan 2008	25,719	65.59%	3,908	9.97%	9,583	24.44%
Dec 2012	55,341	76.13%	5,919	8.14%	11,430	15.72%

Proportions of total number of Gene Ontology relations for Biological process (BP), Cellular components (CC) and Molecular functions (MF) between January 2008 and December 2012.

At this point, our results confirm the initial impression by OnEX that the Gene Ontology complexity increased monotonously as a whole as well as for its three branches, and that BP was the branch with the fastest growth, which explained why CC and MF were proportionally decreasing.

### Variations of connectivity

The number of relations increased, but so did the number of classes. We investigated whether the number of relations increased proportionally more (the graph became denser) or less (the graph became more sparse) than the number of classes. The previous results indicate that between January 2008 and December 2012, the number of relations increased proportionally more than the number of classes for BP, whereas both number increased by similar proportions for CC and MF. We wanted to know if this trend was regular and uniform for the three relations is a, part of and regulates.

**Figure 3:**
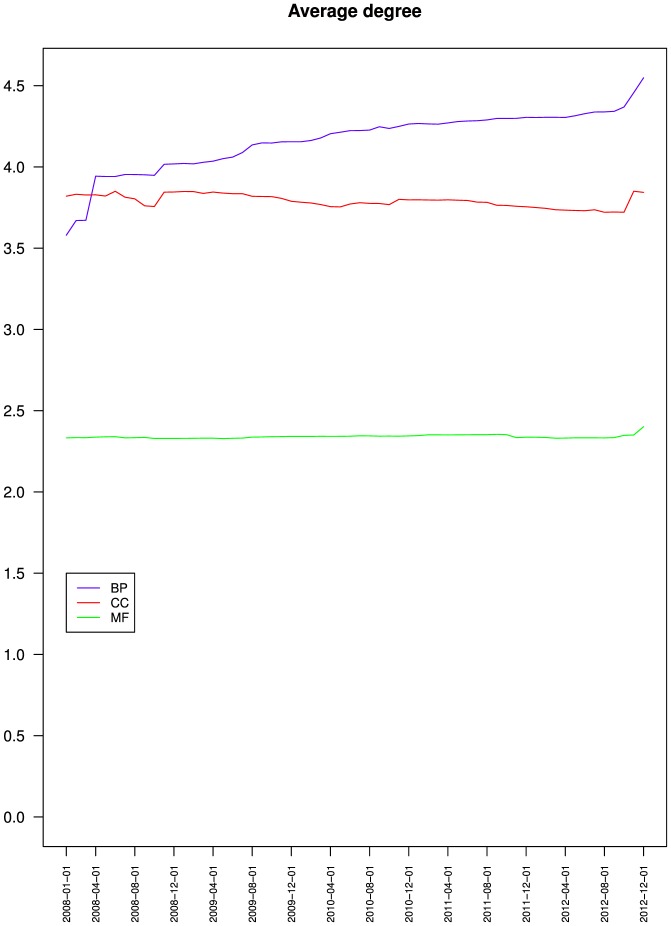
Evolution of the average degree of the nodes of the three branches of the Gene Ontology. Biological process (BP), Cellular component (CC) and Molecular function (MF).

Figure 3 presents the evolution of the average degree of a node for BP, CC and MF. It shows that the average degree of a node was around 4 for BP and CC, and around 2.3 for MF.

Figure 3 also shows that over time, the average degree of a node increased monotonously for BP, decreased slightly for CC with some local variations and a sharp increase in November 2012, and remained stable for MF, which completes the previous observations.

**Figure 4:**
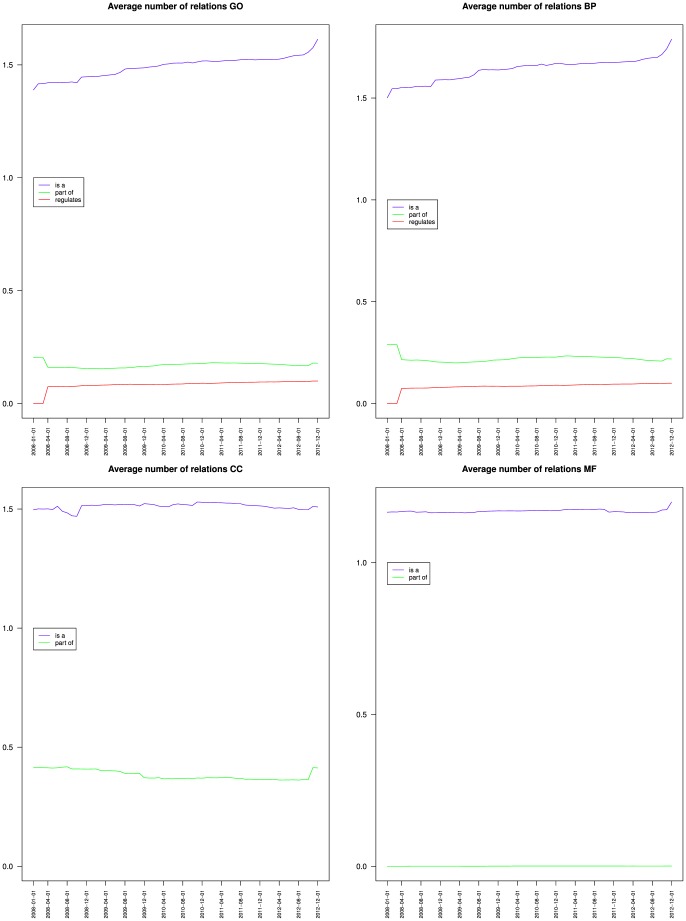
Contributions of the is a, part of and regulates relations to a node's average degree for the Gene Ontology (top left) and its three branches Biological process (top right), Cellular component (bottom left) and Molecular function (bottom right).

Figure 4 and Table 2 present the contributions of the is a, part of and regulates relations to a node's average degree. It shows that the average number is a associated to a node increased for BP but remained stable for CC and MF. The average number of part of associated to a node decreased for BP, was stable for CC and increased slightly for MF. The average number of regulates associated to a node increased for BP.

Overall, these results indicate (1) that GO branches had different connectivity and different variations of connectivity, and (2) that inside a branch the various relations also had different variations.

### Variations of hierarchy

**Figure 5:**
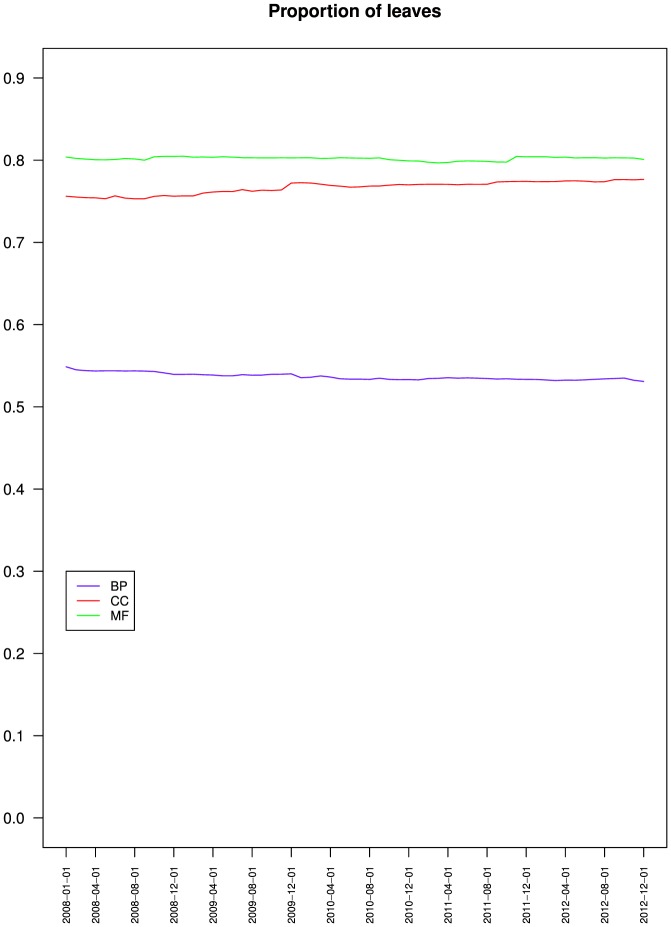
Variations of the proportion of leaves for the Gene Ontology three branches. Biological process (BP), Cellular component (CC) and Molecular function (MF).

Figure 5 presents the variations of the proportion of leaves for GO and its three branches. It shows that the proportion of leaves decreased for BP from 55% to 53.1%, increased for CC from 75.5% to 77.7% and remained stable for MF around 80%. The three branches had different proportions of leaves and different variation patterns. This suggests that the new classes added to BP mostly belong to the intermediate levels of the taxonomy, whereas those added to CC and MF were mostly leaves (maintaining a proportion of 70% to 80% of leaves as the number of classes increases requires that 70% to 80% of the new classes are also leaves).

**Figure 6:**
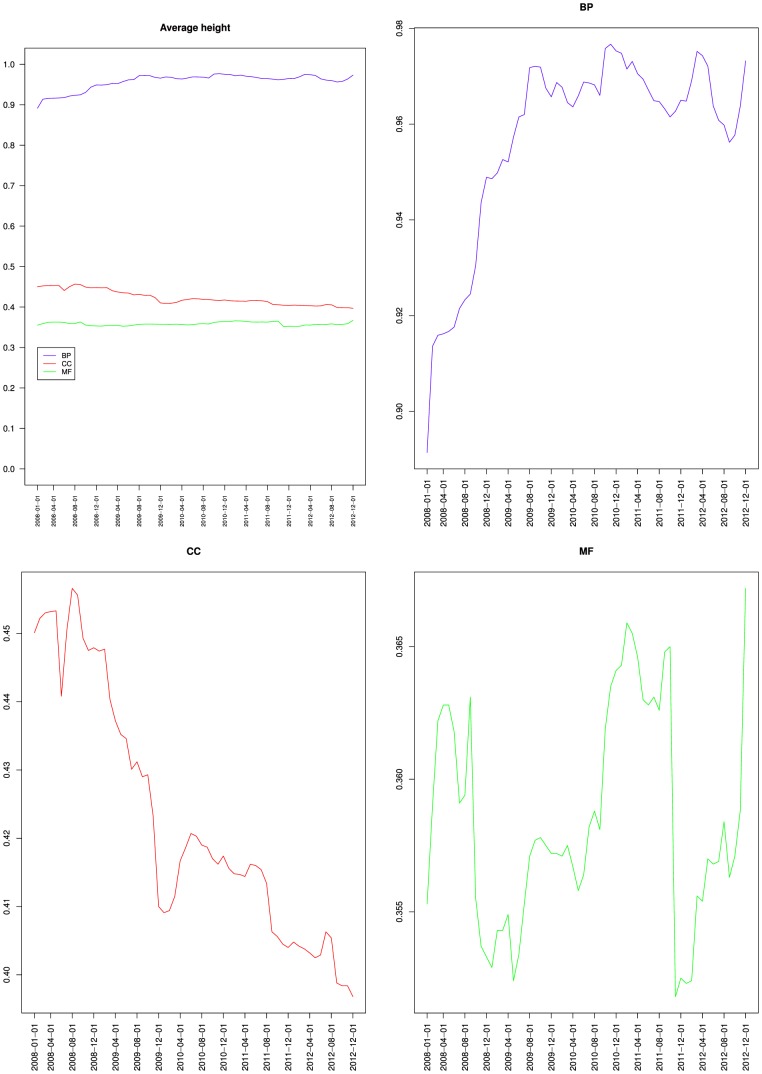
Variations of the average height of the nodes from the Gene Ontology: together (top left), Biological process (top right), Cellular component (bottom left) and Molecular function (bottom right).

Figure 6 presents the variations of the average height of the nodes from GO and its three branches. It shows that nodes average height increased globally for BP but has been mostly stable since June 2009, decreased for CC and remained mostly stable for MF, which confirms the indications of Figure 5.

**Figure 7:**
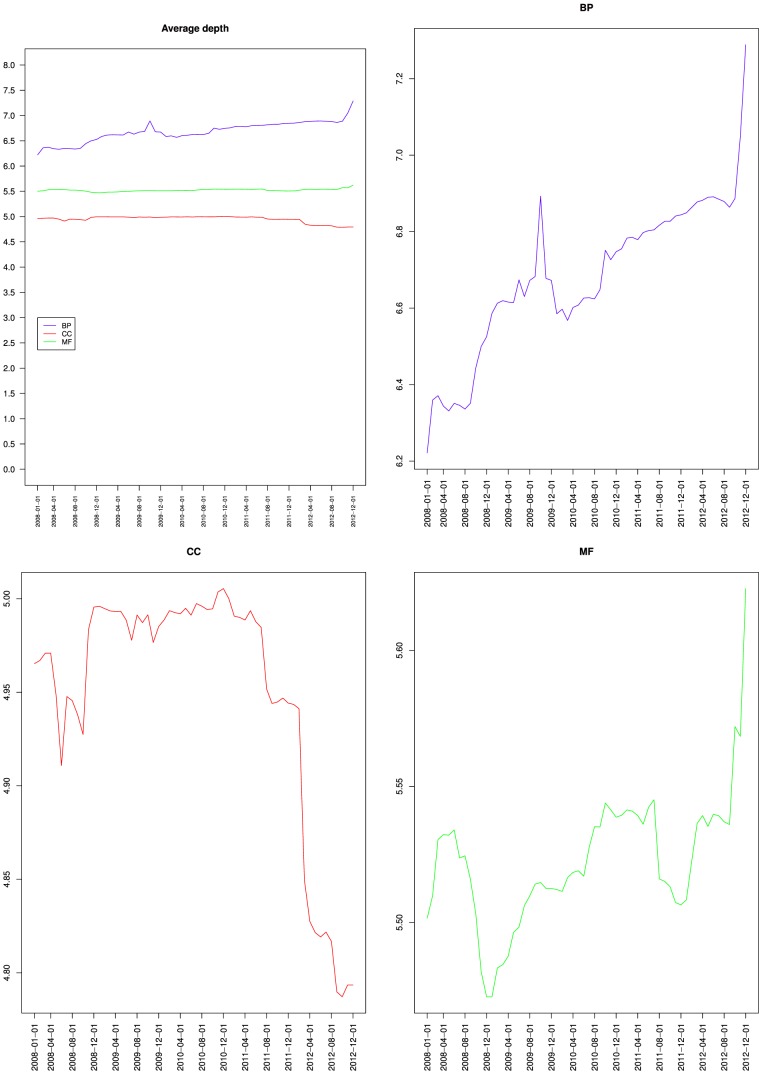
Variations of the average depth of the nodes from the Gene Ontology: together (top left), Biological process (top right), Cellular component (bottom left) and Molecular function (bottom right).

Table 2 shows that the maximum depth increased slightly from 13 to 16 for BP, remained at 10 for CC and increased from 14 to 15 for MF. Figure 7 presents the variations of the average depth of the nodes from GO and its three branches. It shows that nodes average depth increased for BP, and remained mostly stable for MF, which confirms the observations of Figures 5 and 6. The fact that for BP both the average depth and the average height increased reinforces the idea that most of the new BP classes were not leaves (or the average height would have decreased), but were parents or ancestors of leaves (because the average distance to a leaf was 0.97) at least 7 edges away from the root (because the average distance to the root increased from 6.2 to 7.3). Figure 7 also shows that the average depth remained mostly stable for CC until March 2012, when it dropped. Together with Figures 2, 5 and 6, this indicates that the new classes added to CC were mostly leaves, and were siblings of existing leaves so that depth was not affected. The March 2012 drop cannot be explained by the variations of number of classes nor of relations or leaves. This suggests some reorganization of the classes hierarchy.

Figures 5, 6 and 7 also compare the relative values of BP, CC and MF proportion of leaves, average height and average depth. The three metrics reflecting the semantics of the ontology exhibited a similar pattern with CC and MF having similar values compared to BP. This should be contrasted with connectivity metrics from Figure 4 where BP and CC had similar average degree values, compared to MF. Interestingly, CC was similar to BP from a connectivity point of view, and similar to MF from a semantic structure point of view. The similar connectivity of BP and CC is reinforced by the fact that both rely on is a and part of relations, whereas MF almost exclusively uses is a (Table 2).

### Comparison with random ontology enrichment

The previous results about the local variations of node connectivity and the global variations of the graph structure showed some fairly monotonous trends for BP, CC and MF. We investigated if these trends were the result of the sole increase of classes and relations. We studied if the previous metrics could discriminate randomly-generated ontology modifications from genuine ones. Table 5 presents the variation of the number of classes and relations between the February and March 2010 versions of the GO, and the average of these metrics on the fifty simulated ontologies.

**Table 5:** Simulated evolution of the three Gene Ontology branches between February and March 2010.

	BP			CC			MF		
	Feb. 2010	Mar. 2010	simul.	Feb. 2010	Mar. 2010	simul.	Feb. 2010	Mar. 2010	simul.
Nb. classes	18,149	18,544	18,544	2,643	2,688	2,688	8,670	8,687	8,687
Nb. is a	29,796	30,507	30,507	4,014	4,065	4,065	1,047	1,067	1,067
Nb. part of	3,928	4,090	4,090	979	1,000	1,000	4	7	7
Nb. regulates	1,542	1,580	1,580	0	0	0	0	0	0
Av. depth	6.597	6.567	7.275	4.994	4.993	5.022	5.511	5.517	5.513
Av. height	0.968	0.965	1.104	0.409	0.411	0.433	0.357	0.358	0.360
Prop. leaves	0.536	0.538	0.525	0.772	0.771	0.761	0.803	0.802	0.801

Variations of ontology metrics for Biological process (BP), Cellular components (CC) and Molecular functions (MF) between February and March 2010, compared to the average of fifty randomly-enriched simulations.

Connectivity metrics are based on the average number of relations. Therefore, they were not affected by the simulations.

**Figure 8:**
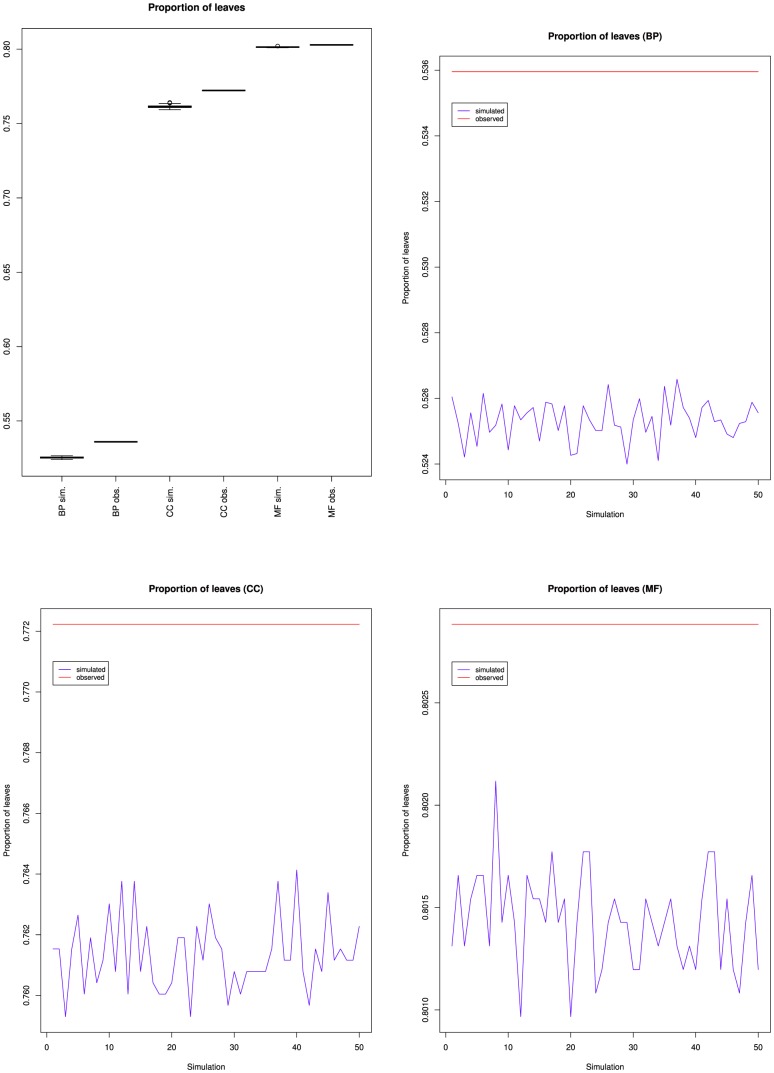
Proportion of leaves for the fifty simulated ontologies, compared to the value for the March 2010 version of the Gene Ontology (red line).

**Figure 9:**
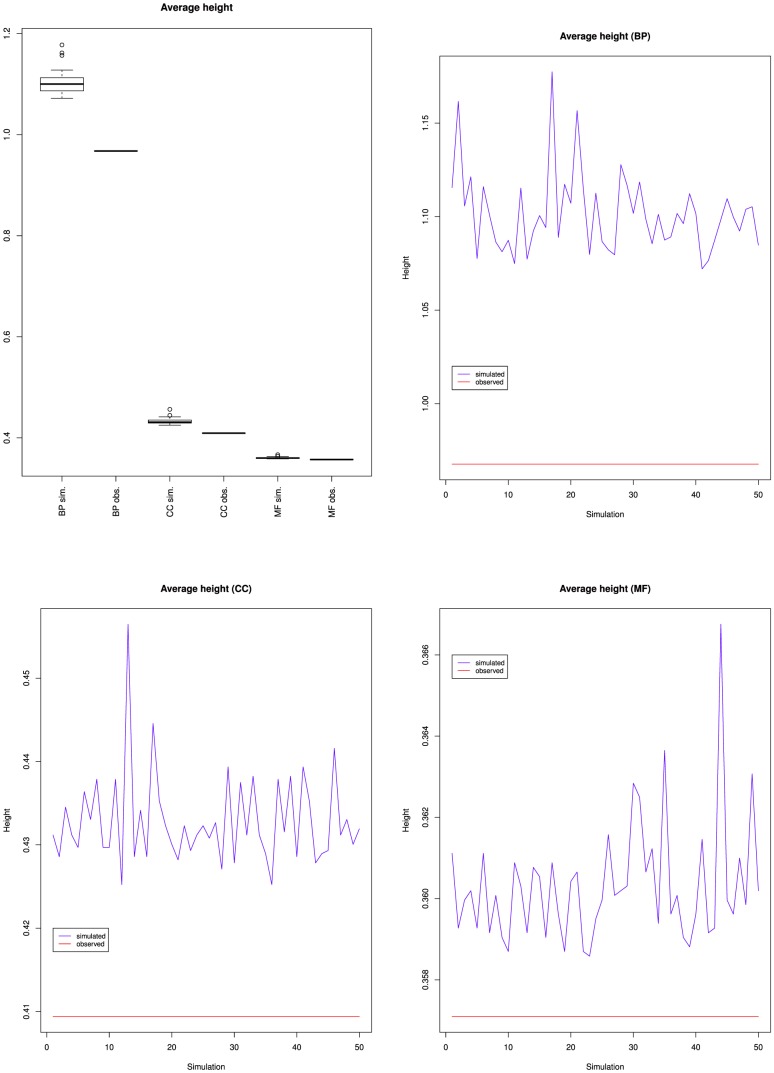
Average classes' heights for the fifty simulated ontologies, compared to the value for the March 2010 version of the Gene Ontology (red line).

**Figure 10:**
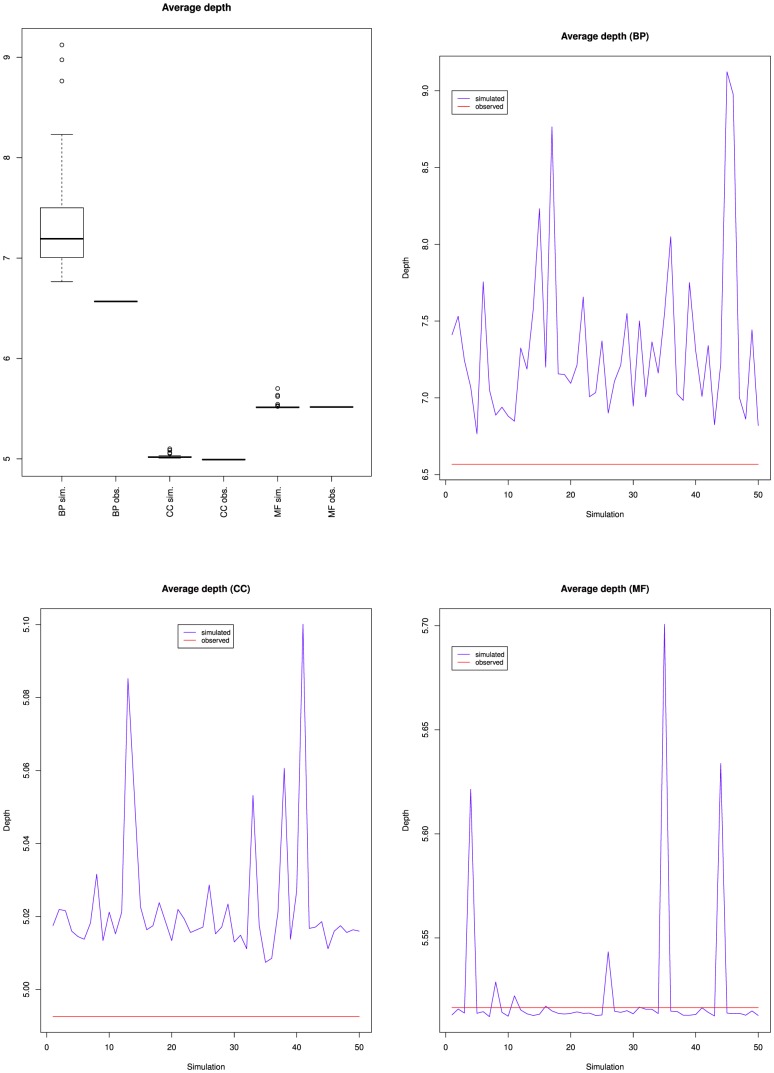
Average classes' depths for the fifty simulated ontologies, compared to the value for the March 2010 version of the Gene Ontology (red line).

Figures 8, 9 and 10 present the proportion of leaves, average height and average depth of the simulations compared to the March 2010 version of GO.

BP simulations had fewer leaves, higher average depths and higher average heights than GO. CC simulations had fewer leaves and higher average heights than GO, but similar average depths. MF simulations had fewer leaves than GO, but similar average heights and average depths.

Table 6 presents the p-values of the Student's t-tests. All the tests showed a statistically significant difference between the simulated and the observed values, except for the average depth in MF. For MF, the fact that the average height increased more in the simulated ontologies than in the March 2010 version of GO, and that the proportion of leaves decreased more in the simulations suggests that the simulated classes were mostly added as non-leaves. The lack of statistically-significant difference of average depth is difficult to interpret, specially because there was a difference of average height. Possible factors are the small number of modifications for MF (but this argument also hold for the other measures), or the structure of MF hierarchy.

**Table 6:** Comparison of the fifty randomly enriched ontologies with the March 2010 version of Gene Ontology.

	BP	CC	MF
av. depth	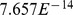	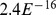	0.1643
av. height	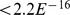	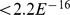	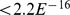
proportion leaves	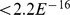	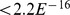	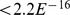

P-value of Student's t-tests comparing the fifty randomly enriched ontologies with the March 2010 version of Gene Ontology.

Together, the random ontology enrichment results confirm that the average depth, average height and proportion of leaves can discriminate randomly-generated ontology modifications from genuine ones. The differences between BP, CC and MF also confirm the previous observations that the three branches have different hierarchical organizations, and different evolutions. The lower number of leaves observed in BP, CC and MF for the simulations were consistent with the higher average heights: if randomly-added classes are not leaves, they are at least one edge away from the leaves; since each branch average height was lower than 1, these classes tend to increase the average height. The difference between BP depth and height variations on the one hand and CC and MF variations on the other hand can be explained by the structural differences between the former and the last two. BP has a smaller proportion of leaves than CC and MF so that randomly-added classes are less likely to be leaves than for CC or MF. Interestingly, Pesquita et al. also observed that for the GO, the refinement of CC and MF occurs mostly via single insertions, whereas in BP, groups of related classes are inserted together [21].

These simulations also confirm that in complex graph structures like ontologies, a small number of changes in the topology can have dramatic consequences on the overall hierarchy. Applications based on approaches such as term enrichment are highly sensitive to such modifications because the annotations are propagated to the ancestors [22]–[25].

## Discussion

In this section, we first survey related GO-specific works. We then discuss the practical applications of our study. Finally, we discuss how our approach can be generalized to other ontologies and other metrics.

### GO-specific approaches

Several studies analyzed the evolution of the GO from different perspectives.

Park et al. developed visualization methods based on a color-coded layered graph to highlight the changes between two versions of GO [26]. Hartung et al. improved the idea with CODEX, that determines a compact diff based on semantic changes [27]. Both approaches focus on change visualization but leave the interpretation of the modifications to the user.

Leonelli et al. characterized the reasons of the changes. They identified five circumstances warranting changes in the GO by curators: (1) the emergence of anomalies within GO; (2) the extension of the scope of GO; (3) the divergence in how terminology is used across user communities; (4) new discoveries that change the meaning of the terms used and their relations to each other; and (5) the extension of the range of relations used to link entities or processes described by GO terms [28]. They focus on improving the way the GO represents biological knowledge but leave the determination of the quality change to the curators and do not measure it.

Köhler et al. proposed a systematic method to analyze the quality of terms definitions [29]. Verspoor et al. developed a transformation-based automatic clustering method for detecting similar terms that use different linguistic conventions [30]. Both approaches focus on the classes names or textual definitions but do not consider the relations among the classes. Mungall et al. proposed an automatic reasoning-based approach using logical definitions for classes and mappings to external ontologies that detects potentially missing and incorrect classes and relationships [31]. It should be noted that even if logical definitions are assigned to all new regulation classes as of January 2010, processing all the previous classes is an ambitious ongoing task. Alterovitz et al. proposed an information theory-based approach to automatically organize the structure of GO and optimize the distribution of the information within it [32]. Faria et al. proposed an association rule-based algorithm for identifying implicit relationships between molecular function terms [33]. Other works focused on the quality of terms definitions [29] and on the detection of semantic inconsistencies of gene annotations [34]. Gross et al. studied to what extent modifications of the GO and of gene annotations databases impacted the result of term enrichment analyses that describe experimental data by sets of GO terms [35]. They demonstrated that the “changes are unequally distributed and cluster in regions representing specific topics”. Interestingly, they also observed that these changes do not necessarily modify the result of term enrichment analyses since the terms are often semantically related. Our results indicated that for BP, most modifications occurred deep into the hierarchy, so it is also possible that term enrichment analyses return sets of more general GO terms that are more stable. Loguercio et al. proposed a task-based approach to examine the completeness and utility of GO annotations for gene enrichment analysis [24]. It should be noted that over time, both gene annotations (i.e. the set of GO terms associated to gene products) and the GO itself evolve simultaneously. They focused on the quality of annotations, whereas we focused on GO proper. Moreover, as stated in the background section, the metrics of complexity we used are intrinsic values that are task-independent.

Ceusters performed an extensive evolutionary terminology auditing [36] of the GO between 2001 and 2007 for measuring to what extent the structure of a terminology mimics reality. This avoids mistakes, some of which are not eliminated by automatic reasoning. He reports that the quality of the BP, CC and MF branches of the GO increased continuously over time, with MF having consistently the highest quality. He also observed a 'high correlation (0.95) between the increase in size of the GO as a whole and the quality scores'. This should be contrasted with our results (admittedly over a different period) showing that the complexity increased for BP, decreased slightly for CC and remained stable for MF.

Pesquita and Couto proposed a semi-automatic approach for change capture, i.e. the identification of the areas of an ontology that need to be changed [21]. They applied it to 6-months spaced snapshots of the GO over the 2005–2010 period to study whether their framework could predict the portions that would be extended. Their focus was on the analysis of the new classes and relations. It relied on (1) the depth of new classes, (2) the number of new classes that are children of (former) leaves, and (3) the number of new classes that are children of existing classes vs. of newly added classes. This allowed to determine the general direction of refinement (i.e. if new classes provide a finer description or cover a new domain) and whether new classes are inserted individually or as parts of a new branch. They observed that in BP, CC and MF, the majority of new subclasses are added as children of non-leaf classes. They also observed that the refinement of CC and MF occurs mostly via single insertions, whereas in BP, groups of related classes are inserted together. Their observations are compatible with our results. It should be noted that their approach focuses on the analysis of the features of the new classes, whereas we studied BP, CC and MF globally and focused on the consequences of the changes (not just the additions) on the ontology itself. Therefore, we believe the two approaches complement each other.

### Practical applications

The main consequences of our results concern people maintaining GO annotations, as well as developpers of data analysis methods based on the GO.

The regular addition of leaves or of classes close to leaves for BP and CC indicates that over time, more precise terms were being added to the GO hierarchy. Some of the former annotations that refer to the parents of these new classes could be transferred to the new classes. Because of the rule of annotations propagation to the ancestors, the former annotations would remain valid, but this would result in a gain in annotation precision. With the OnEX web application, Hartung et al. proposed a mechanism capable of semi-automatic migration of outdated annotations [4]. Our results indicate that the addition of new low-level classes (mostly for BP and CC) has potential implications on former annotations, whereas higher level classes (mostly for MF) represent previously undescribed topics. The latter situation is not compatible with the OnEX semi-automatic migration approach. Ideally, experts should decide whether these new high-level annotations are suitable for existing entities such as gene products.

The parallel evolution of the GO and of annotations databases has consequences on the results of data analysis studies [37] as well as on the evaluation of GO-based data analysis methods [38]–[40]. Gillis et al. reported that “GO annotations are stable over short period of time”, but also that “genes can alter their functional identity with 20% of gene not matching to themselves (by semantic similarity) after two years” [25]. The direct implication is that all the results of analyses based on the GO should be re-assessed on a regular basis. By showing that complexity increased for BP and CC with the addition of leaves or of classes close to leaves and that MF complexity remained stable with uniform modifications, our study suggests that the conclusions of the previous analyses could remain valid but may actually be improved, although quantifying this assumption would be a separate work. Similarly, the respective performances of GO-based data analysis methods should be re-evaluated on a regular basis.

These metrics could be integrated into at least three kinds of future applications. First, they could easily be integrated into ontology-development tools such as Protégé or OBOEdit. However, not all users may have the need to monitor such metrics. Furthermore, comparing the measures when only a few changes have been made may make it harder to identify general trends. We also computed the measures on daily snapshots of GO from July 2009 to July 2012 and observed successive increases and decreases on all values. The second option would then be to integrate our metrics on top of the ontology version control system. We have seen that computing the measures between commits is not very informative, whereas comparing their evolution between releases (i.e. when the curators judge that a set of commits achieved a meaningful goal) makes more sense. The third alternative would be to integrate our metrics into ontology repositories such as Onex (http://dbserv2.informatik.uni-leipzig.de:8080/onex/or Bioportal (http://bioportal.bioontology.org/). This solution is user-oriented, whereas the second one was curator-oriented.

### Generalization

Our approach relies on classic DAG metrics, none of which is GO-specific. Therefore, our approach is readily applicable to any other ontology. It has the advantage of genericity, but the drawback is that it would probably ignore some ontologies peculiarities (e.g. the positive and negative regulation pattern, which has an impact on the nodes' degree). These would have to be taken into account when interpreting the results.

This argument makes the comparison of the values between ontologies questionable (e.g. to determine thresholds or to provide some qualitative interpretation). We advise to focus on the evolution of measures during an ontology lifecycle.

The next challenge will be to propose new ontology complexity metrics capable of taking into account features of semantically-rich languages such as OWL (http://www.w3.org/TR/owl2-primer/): disjontness between classes, the fact that some relations can be transitive or asymmetric, existential and universal restrictions, etc [41], [42]. The connectivity and hierarchy-related metrics that we presented only cover a limited portion of the meaning conveyed in ontologies. They see ontologies mostly as taxonomies, i.e. a directed acyclic graph of is a relations. Most current ontologies are in the taxonomy category anyway, so taking these additional features into account would probably have a limited impact. However, one can anticipate that these features will gradually gain acceptance as they make ontology maintenance easier, and support more advanced reasoning [43], [44]. Conversely, providing a quantified measurement of their impact on the ontology structure may also help promoting their adoption.

## Conclusion

For the Gene Ontology, the number of classes and relations increased monotonously between January 2008 and December 2012. Considering the three branches of the Gene Ontology (Biological process, Cellular component and Molecular functions) independently gave similar conclusions but revealed different growth rates. Connectivity and hierarchy-related metrics provided additional insights into the ontology complexity. They revealed different patterns in terms of values as well as of evolution.

Graph-related metrics such as the average degree of a node provided additional information about the ontology connectivity. For the Gene Ontology, BP and CC had similar average degrees, superior to that of MF. The analysis of the variations of nodes average degree showed that during the study period, the connectivity of BP nodes increased, while it slightly decreased for CC and remained stable for MF. It also showed that the CC decrease could be attributed to the number of part of relations increasing less than the number of CC classes.

Hierarchy-related metrics such as the proportion of leaves, the average depth and the average height of nodes provided information about the semantics. For the Gene Ontology, CC and MF had similar proportions of leaves, average depths and average heights, that were superior to that of BP for the proportion of leaves, and inferior to BP average depth and average height. The proportion of leaves decreased for BP, increased for CC and remained stable for MF. The nodes average height increased for BP, decreased for CC and remained mostly stable for MF. The nodes average depth increased for BP, remained mostly stable for CC until March 2012 and then decreased, and remained mostly stable for MF. These measures also indicated that most of the classes added to BP were not leaves but were in the lowest part of the hierarchy, whereas most of the classes added to CC were leaves and siblings of existing leaves, and that MF growth was rather uniform. Eventually, hierarchy-related measures could distinguish the actual GO evolution from the random addition and removal of classes and relations.

Overall, for the Gene Ontology, the results showed that the three branches Biological Process, Cellular Component and Molecular Function have to be considered separately when studying the evolution of the Gene Ontology complexity. The number of classes and relations increased monotonously for all branches. Our results show that the changes operated by Gene Ontology curators between monthly releases impact both the ontology size and the ontology complexity. Node connectivity increased monotonously for BP, decreased globally with several local extrema for CC and was stable for MF, with BP and CC having similar profiles compared to MF. Concerning the hierarchy, average depth and average height increased for BP, decreased for CC and was stable for MF, with CC and MF having similar profiles compared to BP. These results indicate that BP was the most dynamic branch which complexity increased, that CC was refined with the addition of leaves providing a finer level of annotations but complexity decreased, and that MF experienced a stable and uniform growth.

## Supporting Information

Spreadsheet S1 **Analysis of the Gene Ontology monthly releases 2008–2012.** (S1-geneOntology-complexityEvolution-monthly.ods) contains the analysis of the sixty Gene Ontology monthly releases between January 2008 and December 2012.
(ODS)

Spreadsheet S2 **Analysis of the simulated random ontologies.** (S2-geneOntology-enrichmentSimulations.ods) contains the analysis of the fifty simulated random ontologies.
(ODS)
